# A tight binding study of electron transport in notched graphene nanoribbons

**DOI:** 10.1038/s41598-025-03707-z

**Published:** 2025-06-01

**Authors:** Mohamed R. Maamoon, A. M. Khalaf, M. Kotb, M. S. Sadeq, Mohammad A. Kher-Elden, Ignacio Piquero-Zulaica, Zakaria M. Abd El-Fattah

**Affiliations:** 1 Physics Department, Faculty of Science, Al-Azhar University, Nasr City, Cairo, 11884 Egypt; 2 Basic Science Department, Faculty of Engineering, Sinai University-Kantara Branch, Ismailia, 41636 Egypt; 3 Centro de Física de Materiales CSIC/UPV-EHU, Manuel Lardizabal 5, 20018 San Sebastian, Spain; 4 IKERBASQUE, Basque Foundation for Science, Plaza Euskadi 5, 48009 Bilbao, Spain; 5 Physics Department, Faculty of Science, Galala University, New Galala City, Suez, 43511 Egypt

**Keywords:** Condensed-matter physics, Electronic properties and materials

## Abstract

Edge corrugated, notched graphene nanoribbons (GNRs) exhibit intriguing electronic properties distinct from their straight counterparts, thereby offering suitable candidates for the exploration of electron transport in future carbon-based nanoelectronic devices. Here, we utilize the tight binding (TB) method to investigate the electronic structure and quantum transport in gulf- and chevron-type notched GNRs. Consistent with earlier TB calculations, we reaffirm that the third-nearest neighbour hopping parameter is responsible for the electronic braiding effect and zero-energy conductance channels in *straight* zigzag GNRs (ZGNRs), here demonstrated for 2ZGNR and 5ZGNR. However, for notched gulf- or chevron-type GNRs, generated by selectively eliminating carbon atoms at either one or both ZGNR edges, the electronic band structures can be radically changed from semiconductor to metallic, with near-Fermi dispersive or flat bands. For the explored asymmetrically notched chevron-type GNRs hosting a metallic flat band at the Fermi energy, unlike straight ZGNRs, the electronic transport was found to depend primarily on the second-nearest neighbour, exhibiting a sharp conductance peak (of 1 unit conductance) at the Fermi energy. This result is found to be generic for all asymmetric chevron-type GNRs, irrespective of the nanoribbon width, and also for edge-notched armchair GNRs hosting similarly metallic flat bands. For the metallic symmetrically notched chevron-type GNR, however, the near-Fermi dispersive bands lead to multiple conductance channels around the Fermi energy, with fine structure dependence on the number of hopping parameters utilized. These results are analyzed with respect to the spatial distribution of the metallic states and how they transverse across the ZGNR leads. The present study should have large implications on the exploration of electronic transport in carbon-based nanoelectronic devices.

## Introduction

Since the pioneering work by Novoselov *et al.*^1^ on the exfoliation of a single graphite layer, namely graphene, research on two-dimensional (2D) materials has rapidly developed. Among currently existing potential 2D materials^2–6^, graphene - the atomically thick planar 2D sheet of carbon atoms arranged in a honeycomb lattice^7^—remains a promising frontier candidate for diverse applications including carbon-based nanoelectronics, given its unique $\pi$ electronic structure^8^. For operational nanoelectronic devices based on graphene, a band gap engineering is essential, for which different approaches have been envisioned, including the symmetry breaking of carbon sub-lattices^9^, but most efficiently through the fabrication of 1D and 0D graphene nanostructurues, such as graphene nanoribbons (GNRs), quantum dots, nanographene structures (e.g., triangulene), etc.^10–15^. GNRs, in particular, facilitate the generation of distinct electronic structures and confinement-induced energy gaps that are tunable through the width of the nanoribbon and the edge termination^16–18^. Likewise, edge-corrugated GNRs, in the form of chevrons or zigzags, for example^19–25^, were found to induce exotic electronic properties such as topological end states, thereby widening the range of applications of graphene-based topological materials^26–28^. While the edge-corrugated GNRs in the form of chevrons made out of pure carbon atoms (pristine (undoped) graphene nanoribbons (p-GNRs)) possess unique electronic and magnetic properties^29^, the fabrication of nitrogen-doped graphene nanoribbons (N-GNRs) led to promising heterostructures physically identical to the traditional p-n junctions and suitable for photovoltaics applications^30^. This breakthrough motivated further theoretical and experimental investigations^31,32^. Also, the fabrication of the nitrogen-rich graphene nano chevrons led to promising structures with electronic properties which may be tuned both by choosing the precursor and the way of linking them to produce the periodic structure^20^. These nanoscale carbon structures are expected to exhibit appealing quantum transport effects and applications^33^, such as quantum blockade^34^, single electron transistor based on a width-modulated GNRs^35^, graphene-based charge detectors^36^, etc.

For the exploration of the distinct electronic properties of these graphene-based nanostructured materials, the first and higher nearest neighbour tight binding (TB) method has played a central role, given its efficient computational time and transparency to graphene physics as offered by the control over the hopping parameters^37,38^. For instance, TB band structure calculations performed for narrow - a couple of phenyl ring wide - zigzag GNRs (ZGNRs) revealed the presence of valence and conduction band braiding for the edge state^39^, leading to a number of incommensurate Dirac points, in agreement with density functional theory (DFT) and electron plane wave expansion (EPWE) calculations^39,40^. Following TB analysis, it was revealed that the reported braiding effect is only relevant for narrow ZGNRs and is solely induced by the *third*-nearest neighbour hopping ($t''$). Interestingly, this peculiar braiding phenomenon was also shown to open new conductance channels at/near the Fermi energy in TB transport calculations, where the conductance magnitude and the spanned energy range are tunable with the ZGNR’s width^39^. We note that these zero-energy conductance channels, which assume integer multiples of the quantum conductance (2$e^2$/*h* unit) for these *narrow* and *straight* ZGNRs are not present for first nearest neighbour (*t*) or second-nearest neighbour ($t'$) TB calculations^39^.

Edge-corrugated ZGNRs, notched in the form of gulf, chevron, or zigzag, for example, have shown to exhibit electronic properties distinct from their straight ZGNR counterparts, revealing semiconducting or metallic band structures according to EPWE calculations^40^. In this work we offer alternative ways to corrugate ZGNRs either to open a band gap or to induce a near-Fermi dispersive or flat bands. These induced bands should have large implications on quantum transport and thus, these notched ZGNRs could be used in nanoelectronic devices. In addition, we explore the effect of nearest neighbor hopping parameters, in TB calculations, on the electronic properties and electron transport in ZGNRs notched in the form of a gulf or chevron. Specifically, we do not observe hopping-dependent braiding effects in the explored structures, irrespective of the number of nearest neighbour hopping parameters utilized. Importantly, unlike the conductance of straight ZGNRs, we find that the *second*-nearest neighbour ($t'$) is solely responsible for the conductance in these notched nanoribbons leading to electron transport, of one unit quantum conductance, in asymmetrically notched chevron-type ZGNRs featuring flat band at the Fermi energy. This sharp zero-energy conductance goes to zero for $t' = 0$, even when $t''$ is turned on. We attribute this distinction between straight and notched ZGNRs to the spatial distribution of the edge metallic states at the GNR-lead interface, which imposes some constraints on the hopping parameters required to allow for electron transmission. These findings can be generalized to include all chevron-type ZGNRs, irrespective of their width, as well as armchair GNRs (AGNRs) hosting metallic flat bands induced by specific edge corrugation, as demonstrated here for the sawtooth AGNR^41^.

## Results and discussion

**Fig. 1:**
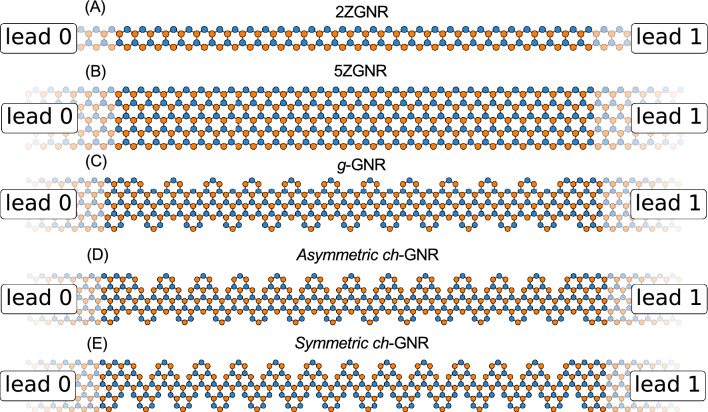
**TB models of selected straight and notched GNRs with leads attached.** TB atomistic structural models for straight 2ZGNR (**A**) and 5ZGNR (**B**) as well as different edge-corrugated polymers, namely gulf-type *g*-GNR (**C**), chevron-type *asymmetric* *ch*-GNR (**D**), and *symmetric* *ch*-GNR (**E**). Two ZGNR leads (lead 0 and lead 1) are attached at the termini of each structure. The leads in (**A**) are 2ZGNR and in (**B**–**E**) are made of 5ZGNR.

Figure 1 depicts the TB atomistic models for selected straight and edge-notched ZGNRs attached, at both ends, to metallic ZGNR leads for conductance calculations. In Fig. 1A,B the atomic structures of typical straight 2ZGNR (A) and 5ZGNR (B) are given, each attached at their termini to ZGNR leads (lead 0 and lead 1) of the same type/width, thereby mimicking the case of infinite ZGNRs. By selectively removing carbon atoms at both edges of the 5ZGNR, we arrive at the notched nanoribbon structure, depicted in Fig. 1C, which we shall call hereafter *gulf-type* GNR (*g*-GNR). This gulf-type structure has been experimentally synthesized utilizing molecular deposition onto the catalytic Au(111) surface^42,43^. Despite the well-known metallic edge state of the parent 5ZGNR, this corrugated nanoribbon was found to exhibit a semiconducting electronic structure with a wide band gap experimentally measured by scanning tunneling spectroscopy (STS) and supported by DFT calculations^42^.

For the forthcoming conductance calculations, the *g*-GNR—and in fact all other edge corrugated structures discussed next—are attached to 5ZGNR leads given their same effective width. To further explore different viable edge termination scenarios and their impact on the electronic band structure and subsequent electron transport, we envisioned breaking the edge symmetry by removing additional carbon atoms at either side of the *g*-GNR structure to obtain the new asymmetric polymer depicted in Fig. 1D, which we call hereafter *asymmetric* chevron GNR (*ch*-GNR). Finally, symmetrizing the previous chevron nanoribbon, by eliminating an additional carbon atom at the other polymer side, we generate the *symmetric* chevron *ch*-GNR shown in Fig. 1E, which has the same effective arm width as the 2ZGNR. This chevron-type polymer is expected to feature a dispersive metallic state near the Fermi energy following earlier EPWE calculations^40^. For reliable conductance simulations, sufficiently large GNRs, consisting of multiple unit cells, are used in order to capture the electronic characteristics of infinite polymers.

**Fig. 2:**
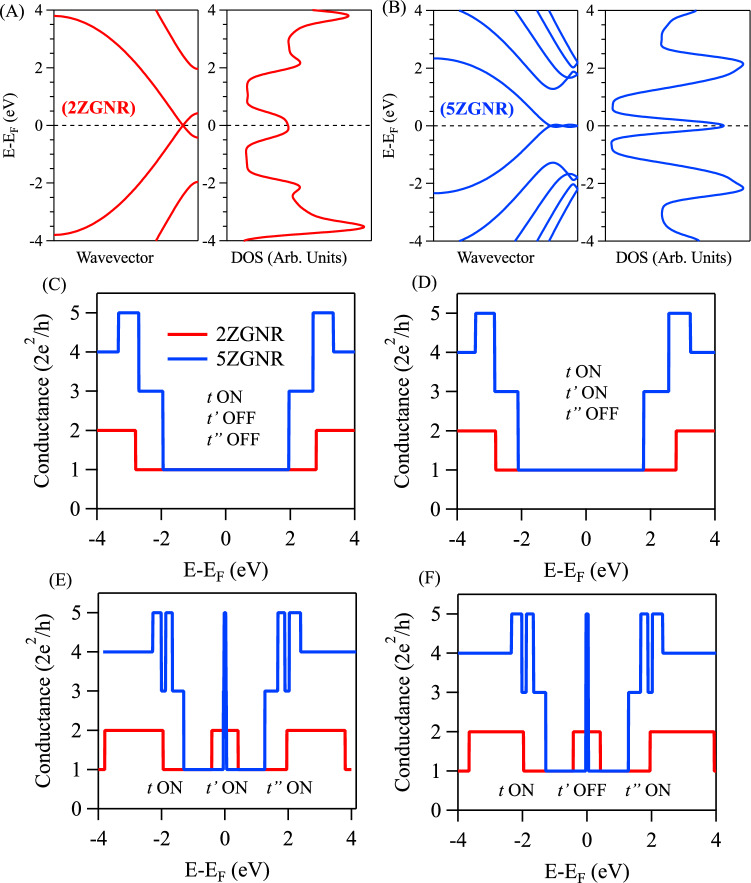
**Electronic structure and transport in straight and narrow ZGNRs.** (**A**, **B**) TB band structures for 2ZGNR (**A**) and 5ZGNR (**B**) and the corresponding DOS shown on the right. The three hopping parameters are turned on in these calculations as evident from the observed band braiding effect. (**C**–**F**) Comparative conductance spectra between 2ZGNR (red) and 5ZGNR (blue). The conductance spectra were calculated at different situations for the hopping parameters as follows: (**C**) only the first-nearest neighbour is on; (**D**) the first- and second-nearest neighbour are on, (**E**) all hopping parameters are on up to the third-nearest neighbour, and (**F**) when the first- and third-nearest neighbour are on (the second hopping parameter is off in this case). New conductance channels open up when the third-nearest neighbour is activated.

In Fig. 2A,B we present the TB electronic band structures and density of states (DOS) for the straight and infinite 2ZGNR and 5ZGNR, utilizing three hopping parameters (*t*, $t'$, and $t''$) into the TB Hamiltonian given in Eq. (1) (see Theoretical Methods section). The values of the hopping parameters are taken from Ref.^39^ and are listed in Table 1. The band structure of 2ZGNR (Fig. 2A, left) exhibits a near-Fermi metallic edge state with strong valence-conduction band braiding ($\sim$ 0.8 eV band width at zone boundary) and a single incommensurate Dirac point, in agreement with earlier TB calculations^39^. The band braiding effect is also evident in the calculated DOS (Fig. 2A, right) showing up as a broad DOS feature around the Fermi energy. Likewise, the TB band structure and the corresponding DOS for 5ZGNR are given in Fig. 2B, where the edge state revealed weaker braiding, in addition to a single commensurate and double incommensurate Dirac points, respectively, leading to stronger and sharper DOS feature at the Fermi, consistent with Ref.^39^. We stress that this edge state braiding effect, and the subsequent formation of incommensurate Dirac points, are only present when the third hopping parameter ($t''$) is turned on in the TB model Hamiltonian, otherwise the upper and lower edge state bands are only touching at the Brillouin zone boundary (see Fig. S1). The near-Fermi modulation of the edge states through the hopping parameters should have strong effects on the electron transport, here demonstrated for the devices depicted in Fig. 1A,B. The TB simulated conductance for these nanoelectronic carbon-based devices are depicted in Fig. 2C–F for 2ZGNR (red) and 5ZGNR (blue) using different combinations of hopping parameters in the TB Hamiltonian. When activating the first hopping parameter (*t*) alone, the conductance spectra exhibit the well-known Landauer quantized step structure, shown in Fig. 2C for 2ZGNR (red) and 5ZGNR (blue). Clearly, around the Fermi energy, the spectra exhibit featureless plateaus of one unit conductance (2$e^{2}$/*h*), while higher conductance is achieved at higher energies scaling with the number of momentum-integrated bands. Specifically, the observed conductance features can easily be inferred from band structure calculations by counting the number of bands sampled at each fixed energy, as schematically explained in Fig. S2. Practically, similar conductance behaviour is obtained when both *t* and $t'$ are turned on, as depicted in Fig. 2D. We note that the band structures of 2ZGNR and 5ZGNRs, for single hopping *t* and for both *t* and $t'$ turned on, do not exhibit the braiding effect (see Fig. S1), and thereby, one branch of the edge states contributes to the calculated conductance. When activating all hopping parameters (*t*, $t'$, and $t''$) new conductance channels open up near the Fermi energy, reaching 2 and 5 units conductance for the 2ZGNR and 5ZGNR, respectively, as shown in Fig. 2E. The width of these conductance features is much narrower for the wider 5ZGNR, and is induced by the braiding effect solely triggered by the third hopping parameter ($t''$), where the number of momentum-integrated bands at these energies increases with the ribbon width (see Fig. S2). Even when $t'$ is turned off, the near-Fermi conductance channels are still present, as shown in Fig. 2F, in complete agreement with the detailed TB study performed in Ref.^39^ for a wider range of ZGNRs of even and odd width. The present analysis reaffirms the findings in Ref.^39^ highlighting the importance of the third nearest neighbour hopping parameter when computing the electrical conductance for the straight and narrow ZGNRs. In the following we address whether the third-nearest neighbour hopping is always responsible for a zero-energy conductance in narrow but notched GNRs and if the one-to-one correspondence between band structure and conductance simulations is generally valid. We shall show that the hopping parameters relevant for opening conductance channels at the Fermi do not necessarily require turning on $t''$ and are dependent on the edge structure and, additionally, enumerating the number of bands in band structure calculations need to be complemented with local density of states (LDOS) calculations to monitor the wave function transport at the nanoribbon-lead interfaces.

**Table 1:** First, second, and third hopping parameters values.

t (ev)	$t^{\prime }$(ev)	$t^{\prime \prime }$(ev)
2.8	0.07	0.42

**Fig. 3:**
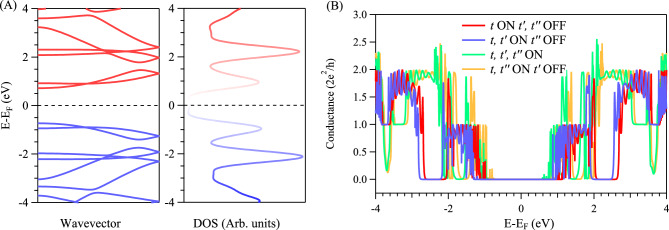
**Electronic structure and conductance in symmetrically notched semiconducting ZGNR:** *g***-GNR.** (**A**) TB calculated band structure (left) and the corresponding density of states (DOS) (right) for the gulf-type GNR, revealing the opening of energy gap between the valence (blue) and conduction (red) bands. (**B**) Conductance spectra with different combinations of hopping parameters. The magnitude of the electrical conductance inside the band gap energy range is zero for all cases.

Figure 3 presents the electronic band structure, DOS, and conductance spectra for the gulf-type *g*-GNR, with the atomistic TB model depicted in Fig. 1C. This *g*-GNR exhibits a clear semiconductor band structure (Fig. 3A, left) with a band gap of $\sim$ 1.44 eV separating the valence (blue) and conduction (red) bands, in agreement with earlier calculations and experimental data^42,43^. Likewise, the semiconducting behaviour is further confirmed from the calculated DOS (Fig. 3A, right) which revealed the absence of any spectral feature around Fermi energy. Although the TB calculations presented in Fig. 3A are performed with the three hopping parameters activated, nevertheless the semiconducting electronic structure is also obtained from first- and/or second-nearest neighbour calculations. Obviously, the presence of such a semiconducting energy gap should lead to zero conductance at the Fermi energy, as shown in the calculated conductance spectra in Fig. 3B, for any combination of the TB hopping parameters, consistent with band structure and DOS calculations. While this pristine *g*-GNR exhibits semiconducting band structure, the presence of structural defects should appear as near-Fermi in-gap defect states typical for most semiconductor materials^44^. In Fig. S3 we show that a single structural defect created at the center of a *g*-GNR, by removing one carbon atom at the top gulf site, produces a Fermi defect state and zero-energy feature in the corresponding DOS. However, the localization of this state at the ribbon’s center does not allow sufficient coupling at the lead-ribbon interface and, thereby, the calculated conductance is practically zero at/near the Fermi energy, despite the presence of a well-defined electronic state with a sharp DOS feature (Fig. S3). In order to open conductance channels at the Fermi energy, coupled periodic array of such structural defects are envisioned, as presented for the *asymmetric* chevron-type *ch*-GNR depicted in Fig. 1D.

**Fig. 4:**
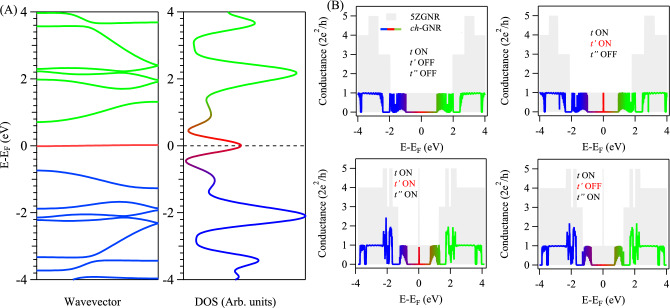
**Electronic structure and conductance in notched ZGNR: Asymmetric** *ch***-GNR.** (**A**) TB calculated band structure (left) and the corresponding DOS (right) for *asymmetric* chevron-type *ch*-GNR, revealing the formation of a metallic flat band and sharp DOS feature (red) at the Fermi energy, inside the energy gap separating the valence (blue) and conduction (green) bands. (**B**) Conductance spectra with different combinations of hopping parameters. A new conductance channel opens up precisely at the Fermi energy solely when the second-nearest neighbour is turned on. The gray backgrounds correspond to the calculated conductance for the straight 5ZGNR at the respective hopping parameters.

In Fig. 4 we depict the TB calculated electronic band structure, DOS, and the conductance spectra for the asymmetrically notched chevron-type ZGNR, namely *asymmetric* *ch*-GNR (Fig. 1D). The band structure in Fig. 4A, left features a flat band at the Fermi energy (red) inside the 1D bulk valence (blue) and conduction (green) bands. This metallic flat band shows up in DOS calculations (Fig. 4A, right) as a well-defined spectral feature at the Fermi energy. As we shall show later, this band corresponds to an electronic state localized at the defective upper edge of the *ch*-GNR. The possible transmission of this Fermi electronic state at the GNR-lead interface can be inferred from conductance calculations, as shown in Fig. 4B. Despite the presence of a metallic band, the conductance spectrum obtained from TB first-nearest neighbour calculations (Fig. 4B (top, left)) exhibits zero conductance at/near the Fermi energy. This behaviour is distinct from the one unit conductance plateau found from the edge state of the straight metallic 5ZGNR (gray) even for single (*t*) hopping parameter (see Fig. 2C). We note that the absence of conductance at Fermi energy is inconsistent with first-nearest neighbour band structure calculations, since the metallic band is still present in these calculations (see Fig. S4). When both *t* and $t'$ are turned on (Fig. 4B (top, right)), a sharp conductance (one 2$e^{2}$/*h* unit) develops at the Fermi energy, assuring the transmission of the edge state through the GNR-lead interface. The same value of Fermi conductance is obtained when the third-nearest neighbour ($t''$) is also turned on (Fig. 4B (bottom, left)). Importantly, when $t'$ is set to zero while *t* and $t''$ are activated, the conductance channel is completely closed, as shown in Fig. 4B (bottom, right). These findings confirm that the emergence of near-Fermi new conductance channels in the asymmetric *ch*-GNR is solely determined by the second-nearest neighbour hopping, in contrast to the straight ZGNRs, where the third-nearest neighbour hopping is the key parameter^39^. Indeed, this finding turned out to be rather generic for all asymmetric chevron-type GNR structures, as we demonstrate for similar edge-corrugated nanostructures notched from narrower and wider ZGNRs, as presented in Figs. S5 and S6, respectively. It is evident from both Figs. S5 and S6, that the second-nearest neighbour parameter is always required for non-zero conductance at the Fermi energy.

**Fig. 5:**
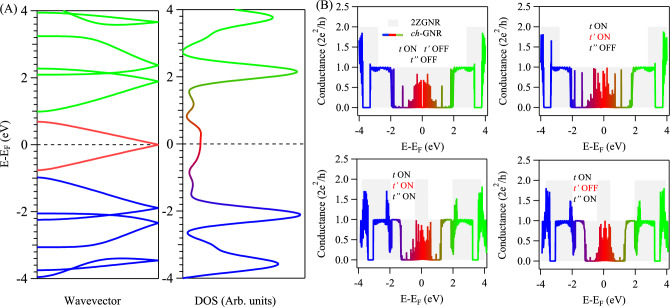
**Electronic structure and conductance in symmetrically notched metallic ZGNR: Symmetric** *ch***-GNR.** (**A**) Calculated band structure (left) and the corresponding DOS (right) for the *symmetric* chevron-type *ch*-GNR, revealing the formation of a metallic dispersive band and broad DOS feature (red) at/around the Fermi energy. The 1D bulk DOS as well as valence and conduction bands are indicated by blue and green colors, respectively. (**B**) Conductance spectra with different combinations of hopping parameters. A new conductance channel opens up spanning the energy window of the metallic band and taking a Gaussian shape centered at the Fermi energy (red). The gray backgrounds correspond to the calculated conductance for 2ZGNR at the respective hopping parameters.

By further eliminating carbon atoms at the lower edge of the metallic and asymmetric *ch*-GNR we arrive at the *symmetrically* notched chevron polymer (*ch*-GNR) sketched in Fig. 1E. This polymer phase exhibits a metallic character as evident from the TB calculated band structure depicted in Fig. 5A, left^40^. Unlike the asymmetric *ch*-GNR, the metallic state (red) is appreciably dispersing ($\sim$ 1.44 eV band width) with a single commensurate Dirac point at the Fermi energy, which manifests as a relatively weak and broad spectral feature in TB calculated DOS presented in Fig. 5A, right. Figure 5B presents the conductance spectra of the symmetric *ch*-GNR structure calculated for different combinations of hopping parameters. The spectra are distinct from the conductance of 2ZGNR (gray background) which has effectively the same arm width of the *ch*-GNR . For all these combinations, the conductance spectra near Fermi energy is characterized by a broad Gaussian conductance profile spanning the energy window of the metallic state band (red) with a maximum value of one unit conductance. The shape of such Gaussian conductance is symmetric when the second-nearest neighbour is turned off (Fig. 5B(top-left and bottom-right)), and is otherwise asymmetric (Fig. 5B(top-right and bottom-left)). For a single-nearest neighbour hopping parameter (Fig. 5B(top-left)), the conductance is precisely zero at the Fermi energy and attains progressively higher values with increasing the number of hopping parameters.

**Fig. 6:**
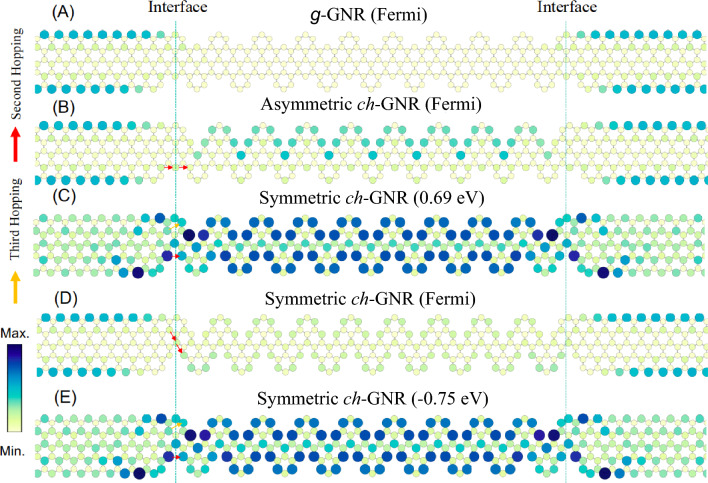
**Local density of states and electron transmission in notched ZGNRs.** LDOS calculations for prototype nanoelectronic devices based on *g*-GNR (**A**), asymmetric *ch*-GNR (**B**), and the symmetric *ch*-GNR (**C**–**E**). All LDOS calculations are performed when the three hopping parameters are turned on. The LDOS calculations in A, B, and D are taken at the Fermi energy. The LDOS in (**C**) is taken at the top of the upper band of the symmetric *ch*-GNR structure, i.e. at $\sim$ 0.69 eV, while the LDOS in (**E**) is at the bottom of the lower band, i.e., at $\sim -0.75$ eV. The red and orange arrows denote the second- and third-nearest neighbour hopping, and the vertical dashed lines mark the GNR-lead interface.

In order to understand the distinct conductance behaviour observed here for the notched GNRs compared to the straight counterparts, particularly the importance of the second- or third-nearest neighbour hopping parameters in computing the electrical conductance, a detailed analysis of the energy-dependent local density of state (LDOS) for the entire device, consisting of the lead-GNR-lead, is essential as addressed in several previous studies^21,45–48^. These LDOS calculations are presented in Fig. 6 for all corrugated GNRs explored here, taken when the three hopping parameters are turned on. Figure 6A depicts the LDOS calculated at the Fermi energy for the semiconducting *g*-GNR-based device. Although LDOS features corresponding to the edge state of the 5ZGNR contacts are present, these are not transmitted across the interface, leading to practically zero spectral weight in the interior of *g*-GNR, consistent with the conductance calculation in Fig. 3. In contrast, the calculated LDOS at the Fermi for the device based on the metallic asymmetric *ch*-GNR (Fig. 6B) revealed the presence of finite LDOS features inside the ribbon, assuring possible transmission across the interface and thereby non-zero electrical conductance as depicted in Fig. 4. We also note that the edge state LDOS feature inside the asymmetric *ch*-GNR structure is clearly localized at the upper edge and, importantly, on one of the sub-lattice types. This explains the absence of conductance at Fermi for first- or both first- and third-nearest neighbour calculations presented in Fig. 4. Therefore, for such asymmetrically notched polymers, the important parameter is the second-nearest neighbour hopping as it allows the propagation of the metallic state within the same type of sub-lattice across the interface, as indicated with the red arrows. In Fig. 6C–E we plot the LDOS for a chevron-GNR-based device taken at the top of the upper metallic band (Fig. 6C), at the Fermi energy (Fig. 6D), and at the bottom of the lower metallic band (Fig. 6E), see the red dispersing bands in Fig. 5A for symmetric *ch*-GNR. For the LDOS taken at both the upper and the lower bands, the intensity is strongly delocalized through the interior of the ribbon allowing finite transmission across the lead interface for any combinations of hopping parameters (see red and orange arrows), consistent with the conductance calculations in Fig. 5. At the Fermi energy (Fig. 6D), however, the LDOS inside the chevron-type *ch*-GNR is much weaker, nonetheless the corresponding states, which are localized on one sub-lattice type, are allowed to couple with the metallic edge states of the 5ZGNR leads via the second-nearest neighbour hopping (red arrows).

**Fig. 7:**
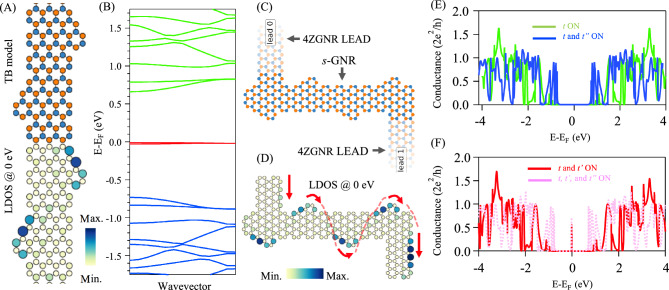
**Electronic properties and transport in sawtooth AGNRs**: *s***-GNR.** (**A**) TB structural model (top) and LDOS at the Fermi energy (bottom) for the *s*-GNR. (**B**) The corresponding electronic band structure showing the presence of weakly dispersing flat bands at the Fermi energy (red) inside the 1D bulk valence (blue) and conduction (green) bands. (**C**,**D**) TB model for *s*-GNR attached to 4ZGNR leads (**C**) and the corresponding LDOS (D) calculated at the Fermi energy. The red arrows indicate the possible electron pathway in conductance simulations. The band structure and LDOS calculations were obtained when the three hopping parameters were activated. (**E**,**F**) Conductance spectra with different combinations of hopping parameters. A new conductance channel opens up precisely at the Fermi energy solely when the second-nearest neighbour is turned on.

Although the GNR structures explored here are all notched from metallic ZGNRs, similar conductance behaviour can be obtained from corrugated armchair GNRs (AGNRs) hosting metallic states inside their 1D bulk band gap^41^. Figure 7A depicts the TB atomistic model for the sawtooth AGNR, namely the *s*-GNR experimentally synthesized on Au(111) surface and explored via DFT and STS in Ref.^41^. The TB-calculated band structure in Fig. 7B agrees well with DFT calculations, revealing two practically flat bands at the Fermi energy (red) inside the semiconducting bands (blue and green). LDOS calculation at Fermi energy in Fig. 7(A, bottom) shows that this metallic state corresponds to edge state coupled across the ribbon, being localized at one sub-lattice type and takes the form of a serpentine pattern traversing through the *s*-GNR width, in agreement with Ref.^41^. To explore the conductance of this metallic state, we simulate the *s*-GNR-lead device shown in Fig. 7C. The calculated LDOS for the entire device at the Fermi energy (Fig. 7D) indicates the possible transmission of the characteristic serpentine pattern through the leads, as indicated by the red arrows. In Fig. 7E,F we present the conductance spectra for the device for different combinations of hopping parameters. In perfect agreement with the asymmetric chevron-type *ch*-GNR in Fig. 4, the Fermi conductance is only possible when the second-nearest neighbour is on (Fig. 7F).

**Fig. 8:**
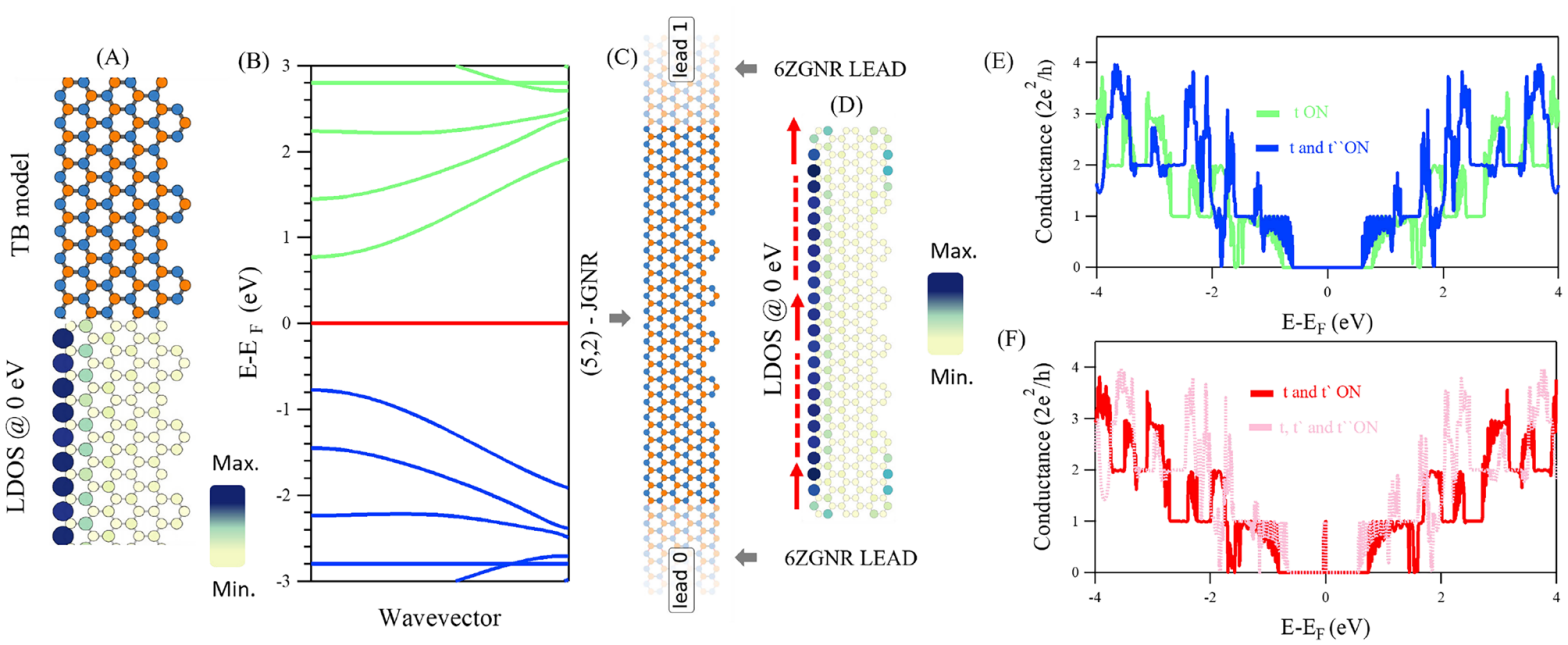
**Electronic properties and transport in Janus ZGNRs:** *J***-GNR.** (**A**) TB structural model (top) and LDOS at the Fermi energy (bottom) for the *J*-GNR. (**B**) The corresponding electronic band structure shows the presence of a metallic state at the Fermi energy (red) inside the 1D bulk valence (blue) and conduction (green) bands. (**C**,**D**) TB model for *J*-GNR attached to 6ZGNR leads (**C**) and the corresponding LDOS (**D**) calculated at the Fermi energy. The red arrows indicate the possible electron pathway in conductance simulations. The band structure and LDOS calculations were obtained when the three hopping parameters were activated. (**E**,**F**) Conductance spectra with different combinations of hopping parameters. A new conductance channel opens up precisely at the Fermi energy solely when the second-nearest neighbour is turned on.

Regarding the unique behaviour of the conductance channel at Fermi energy, particularly from the asymmetric *ch*-GNR and *s*-GNRs nanoarchitectures explored here, where the electrical conductance is dependent on the second hopping parameter, possible nanoelectronic applications may be envisioned such as quantum sensors^49^ and graphene charge detectors^33^, where the conductance peak at Fermi energy can change its energy or magnitude depending on the interaction with the target adsorbates at the conducting edge. These applications may also be integrated with fused asymmetric *ch*-GNRs and *s*-GNRs leading to nanoporous graphene structures, where the pores shall also play a central role in the sensing process, as the edges defining these nanopores are often chemically active. Furthermore, the electron transport here explored can be extended to include the spin degree of freedom in ferromagnetic GNRs. Indeed, GNRs in the form of *Janus* with distinct asymmetric edge configurations, similar to *ch*-GNR, have been recently fabricated, which creates new opportunities for the realization of quantum spin chains and the miniaturization of spintronic devices^50^. The conductance behaviour described above is also observed in such *Janus* GNRs (Fig. 8), further supporting the generality of our findings across experimentally realized GNR architectures.

## Conclusion

We presented a detailed TB study and analysis addressing the correlation between electronic structure and electron transport in atomically narrow and notched ZGNRs as potential candidates for future carbon-based nanoelectronic devices. In contrast to straight ZGNRs in which the near-Fermi conductance is dominated by the third-nearest neighbour hopping, we found that the second-neareast neighbour hopping is more relevant for the conductance in the asymmetrically chevron-type notched metallic GNRs hosting flat bands at the Fermi energy. This finding extends even for wider notched ZGNRs provided they have the same chevron-type edge termination, and also for semiconducting AGNRs notched in the form of sawtooth edge structure. The present study complements earlier transport investigations on straight ZGNRs and opens the door toward the exploration of diverse carbon-based nanoarchitectures for nanoelectronic applications.

## Theoretical methods

We model the extended graphene sheet by the tight binding (TB) model in second quantization formalism using three hopping parameters with using the following Hamiltonian^51^

1 $$\begin{aligned} \begin{aligned}H=&-t\sum _{i,j=NN}c_{iA}^{\dagger }c_{jB}-t^{\prime }\sum _{i,j=N2}\left( c_{iA}^{\dagger }c_{jA}+c_{iB}^{\dagger }c_{jB}\right) \\&-t^{\prime \prime }\sum _{i,j=N3}c_{iA}^{\dagger }c_{jB}+\text { H.c. } \end{aligned} \end{aligned}$$

where *t*, $t^{\prime }$and $t^{\prime \prime }$are the first-, second- and third-nearest neighbour hopping parameters, respectively. $c_{iA}^{\dagger }$ and $c_{iB}^{\dagger }$ are the creation operators which creates an electron at site *i* in sublattice A and B respectively, $c_{jA}$, $c_{jB}$ are the annihilation operators which annihilate an electron from site *j* in sublattice A and B respectively. H.C refers to hermitian conjugate.

We use the open-source Pybinding package^52^ to control and solve Eq. (1) for any combination of hopping parameters and to build the tight-binding Hamiltonian matrix for any desired atomic structure, such as straight or notched ZGNRs and sawtooth AGNRs as explored in this work. For each defined nanoribbon, the band structure and density of states are calculated. The energy broadening used for DOS calculations is 0.2 eV.

By fitting the Hamiltonian in Eq. (1) with the experimental data (or DFT calculations) for the band structure of graphene nanoribbons one arrive to the best fitting parameters. Here we shall stick and follow the parameters taken from Ref.^39^ as given in Table 1.

For computing the electrical conductance we use the following Landauer–Buttiker formula^53^:

2 $$\begin{aligned} G(z)=G_{o}T \end{aligned}$$

here G(z) is the conductance, $G_{o}=2e^{2}/h$ is the quantum conductance unit, and *T* is the transmission function. The transmission function represents the probability of the electron to transmit from the left lead to the right lead, this transmission function can be represented mathematically using Green’s functions of the scattering region and its coupling to the leads as following

3 $$\begin{aligned} T=\operatorname {Tr}\left\{ \Gamma ^{L}(z)G_{c}^{r}(z)\Gamma ^{R}(z)G_{c}^{a}(z)\right\} \end{aligned}$$

where $Tr\left\{ ..\right\}$ is the trace of the product of the retarded $G_{c}^{r}(z)$ and the advanced $G_{c}^{a}(z)$ green’s functions of the scattering region and its couplings to the left $\Gamma ^{L}(z)$ and right $\Gamma ^{R}(z)$ leads, where $z=E\pm i\eta$ and E is the energy where $\eta$ is an infinitesimal real quantity.

The local density of states can be expressed in terms of the retarded Green’s function as following

4 $$\begin{aligned} \operatorname {LDOS}(E)=-(1 / \pi ) \operatorname {Im}\left[ G_c^r(z)_{i i}\right] , \end{aligned}$$

while, the total density of states is given by

5 $$\begin{aligned} \rho (E)=\frac{1}{L} \sum _{i=1}^L \operatorname {LDOS}(E), \end{aligned}$$

where L is the number of the transverse direction sites.

The scattering problem can also be solved using the wave function formulation which is mathematically equivalent to the non-equilibrium Green’s function formalism due to the Fisher-Lee relation^54^.

The electrical conductance using the wave function formulation can be calculated as

6 $$\begin{aligned} G_{a b}=\frac{e^2}{h} \sum _{n \in a, m \in b}\left| S_{n m}\right| ^2 . \end{aligned}$$

where $S_{n m}$ is the scattering matrix and a,b label two leads.

The numerical calculations of electrical conductance were performed using the Kwant code^54^, for the pre-defined atomistic models imported from Pybinding package^52^ and depicted in Fig. 1. The mathematical foundations of Kwant are detailed in Refs.^54–56^.

**Fig. 9:**
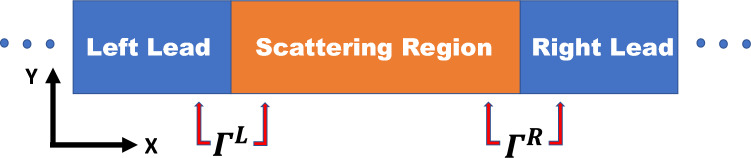
**A schematic diagram for two terminal device (scattering region connected with two infinite leads).**

Figure 9 shows a schematic of the two-terminal device, comprising (i) left lead (source), (ii) scattering region (perfect crystal, no lattice vibrations), and (iii) right lead (drain). In our present study, we assume no electron-phonon interactions. Including such interactions introduces inelastic transmission and reflection with phonon exchange, leading to sub-steps in the quantized conductance whose heights depend on the electron-phonon coupling strength^57^. These effects scale the conductance quantum unit 2$e^2$/*h* but do not affect the principal role of hopping parameters in opening or closing conductance channels near the Fermi level.

## Supplementary Information

Supplementary Information.

## Data Availability

The data presented in this study are available on request from the corresponding authors.
