# Supplementary material for: A tight binding study of electron transport in notched graphene nanoribbons
Source: Sci Rep. 2025 Jun 1;15:19218. doi: 10.1038/s41598-025-03707-z (PMC12127477; doi:10.1038/s41598-025-03707-z)
Supplement: Supplementary file 1 — Supplementary Information. [file 41598_2025_3707_MOESM1_ESM.pdf]

A tight binding study of electron transport in notched graphene nanoribbons

– SUPPLEMENTARY INFORMATION –

Mohamed R. Maamoon,[1,2,*] A. M. Khalaf,[1] M. Kotb,[1] M. S. Sadeq,[2] Mohammad A. Kher-Elden,[1] Ignacio Piquero-Zulaica,[3,4] and Zakaria M. Abd El-Fattah[1,5,†]

[1] *Physics Department, Faculty of Science, Al-Azhar University, Nasr City, E-11884, Cairo, Egypt*
[2] *Basic Science Department, Faculty of Engineering, Sinai University – Kantara Branch, Ismailia, 41636, Egypt*
[3] *Centro de Física de Materiales CSIC/UPV-EHU, Manuel Lardizabal 5, 20018 San Sebastian, Spain*
[4] *IKERBASQUE, Basque Foundation for Science, Plaza Euskadi 5, 48009 Bilbao, Spain*
[5] *Physics Department, Faculty of Science, Galala University, New Galala City, Suez, 43511, Egypt*

(Dated: May 26, 2025)

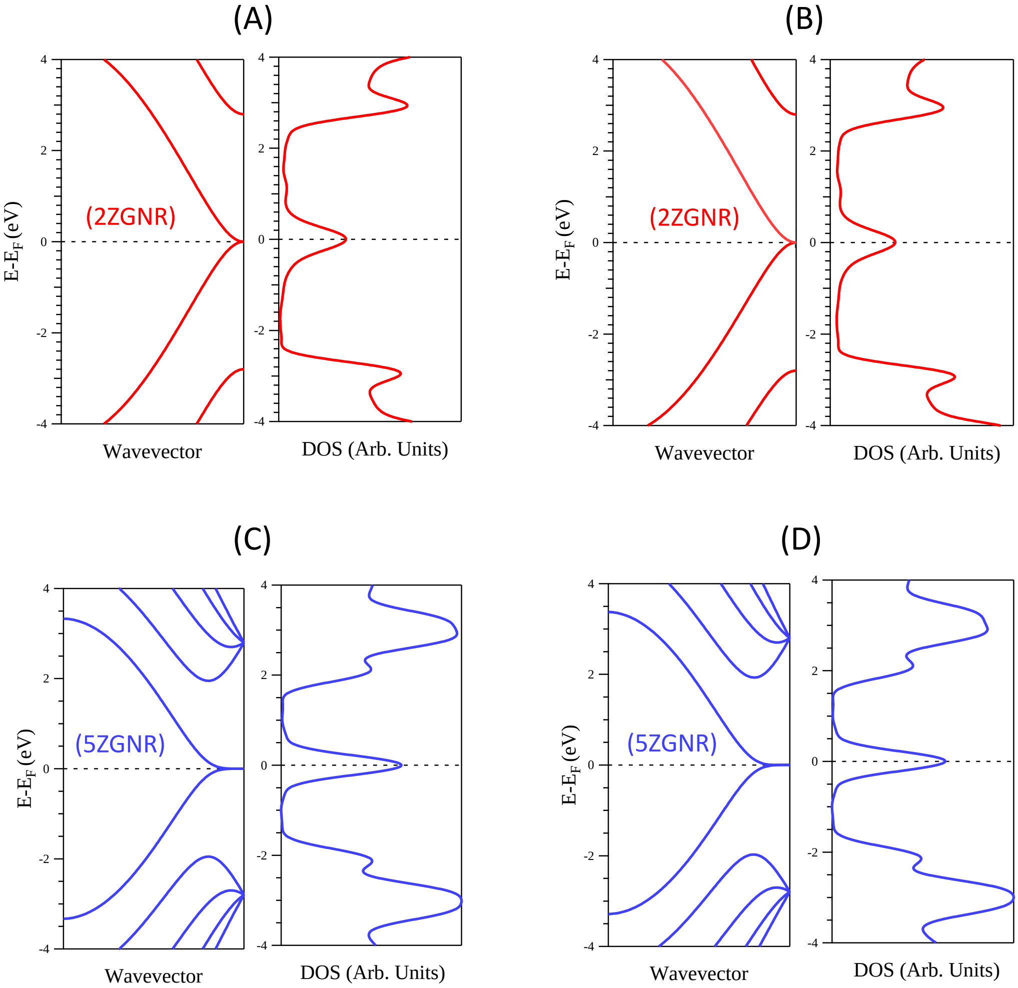

FIG. S1. Band structures and density of states (DOS) for two different zigzag nanoribbons using TB model. (A) Band structure for 2 Zigzag graphene nanoribbon (2ZGNR) (left) and its corresponding density of states (DOS) (right) with activating the first hopping parameter only. (B) Band structure for 2 Zigzag graphene nanoribbon (2ZGNR) (left) and its corresponding density of states (DOS) (right) with activating the first and the second hopping parameters. (C) Band structure for 5 Zigzag graphene nanoribbon (5ZGNR) (left) and its corresponding density of states (DOS) (right) with activating the first hopping parameter only. (D) Band structure for 5 Zigzag graphene nanoribbon (5ZGNR) (left) and its corresponding density of states (DOS) (right) with activating the first and the second hopping parameters.

* Corresponding author: Mohamed.Maamoon@SU.edu.eg, Mohamed.R.Maamoon@gmail.com
† Corresponding author: z.m.abdelfattah@azhar.edu.eg, zakaria.mahmoud@Gu.edu.eg

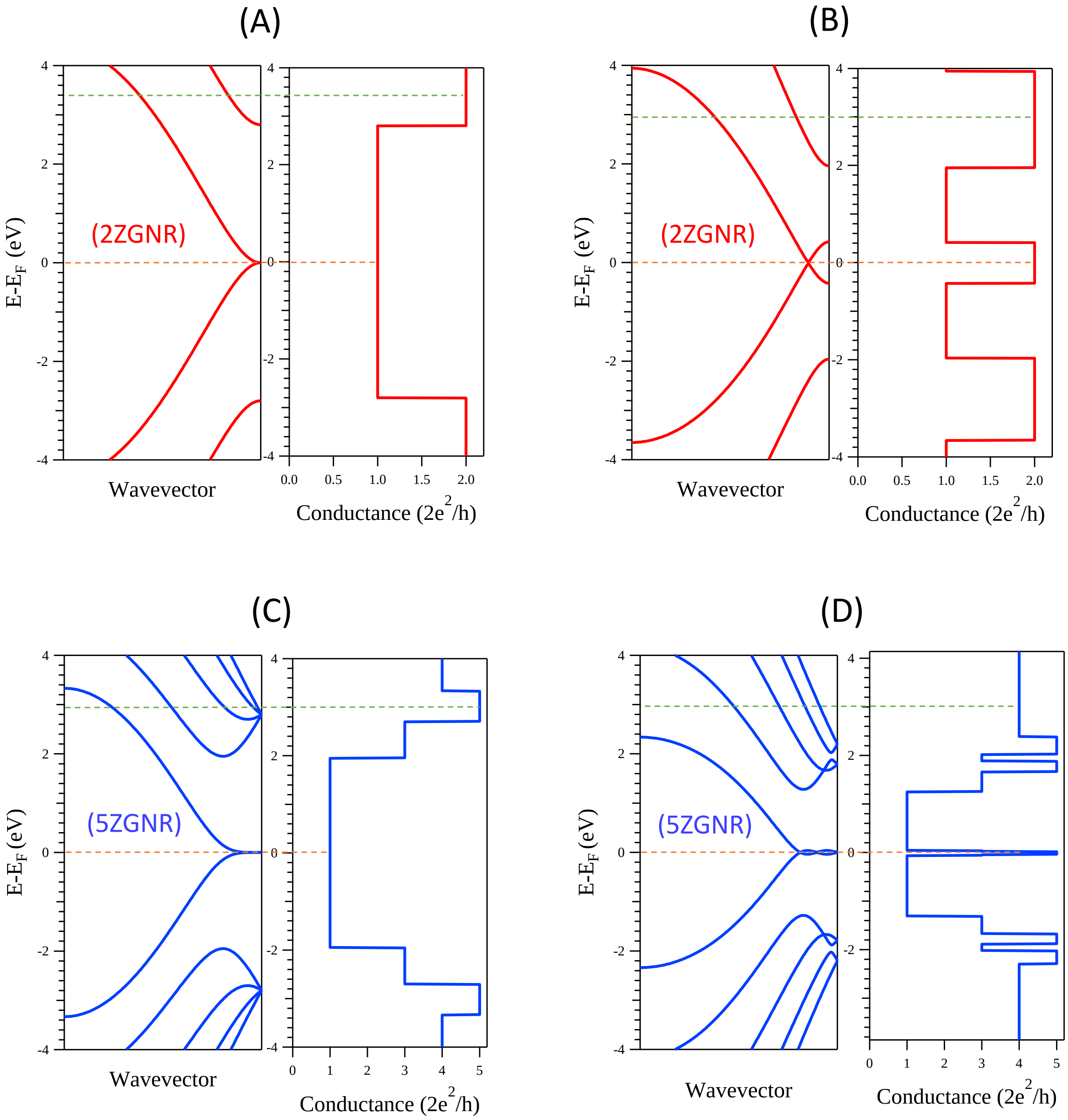

FIG. S2. One-to-one correspondence between band structure (Left) and conductance spectra (Right), The dashed lines indicates to the one-to-one correspondence between band structure and conductance spectra, where the number of bands sampled at each fixed energy give us the corresponding number of conductance units. (A) for 2ZGNR when activating the first hopping parameter only. (B) for 2ZGNR when activating the all three hopping parameters. (C) for 5ZGNR when activating the first hopping parameter only. (D) for 5ZGNR when activating the all three hopping parameters.

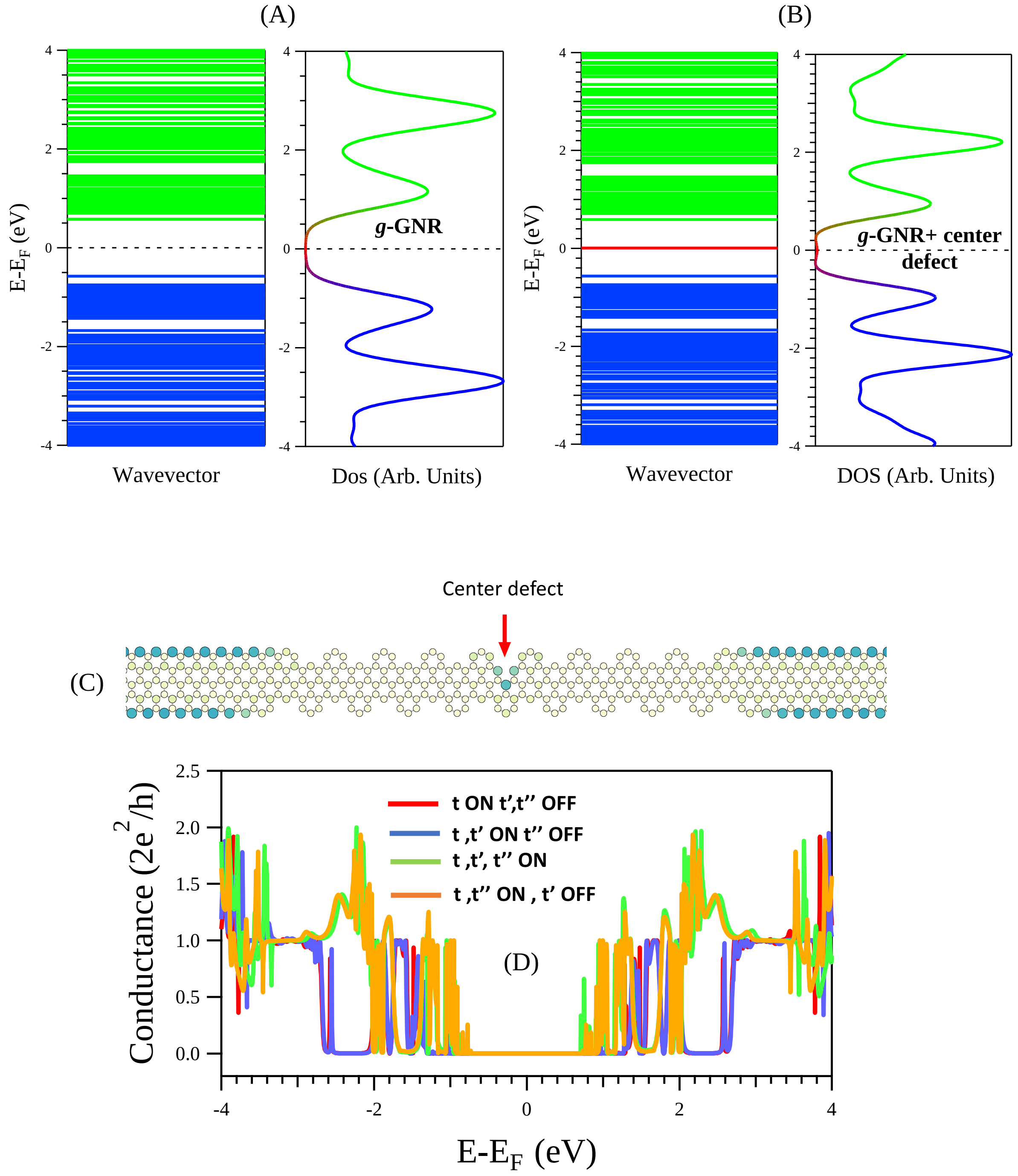

FIG. S3. Electronic structure, density of states (DOS), local density of states (LDOS) and the conductance spectra for pristine *g*-GNR and the center defected *g*-GNR. (A) band structure (left) and its corresponding DOS (right) for the pristine *g*-GNR. (B) band structure (left) and its corresponding DOS (right) for the center defected *g*-GNR. (C) Local density of states for the center defected *g*-GNR. (D) Conductance spectra for the center defected *g*-GNR with different combinations of hopping parameters, showing that the magnitude of electrical conductance at/near Fermi energy is zero for all cases.

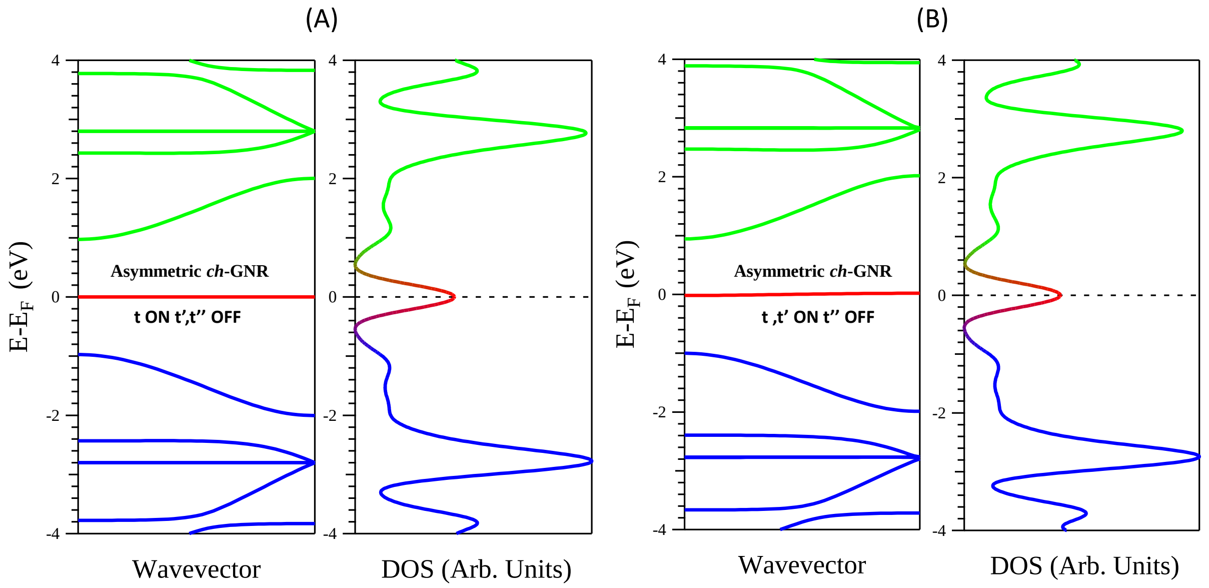

FIG. S4. Electronic structure in asymmetrically notched 5ZGNR: Asymmetric *ch*-GNR. In (A) Band structure (left) and its corresponding density of states (DOS) (right) with using the first hopping parameter (t) only. In (B) Band structure (left) and its corresponding density of states (DOS) (right) with using the first and the second hopping parameters (t' and t'').

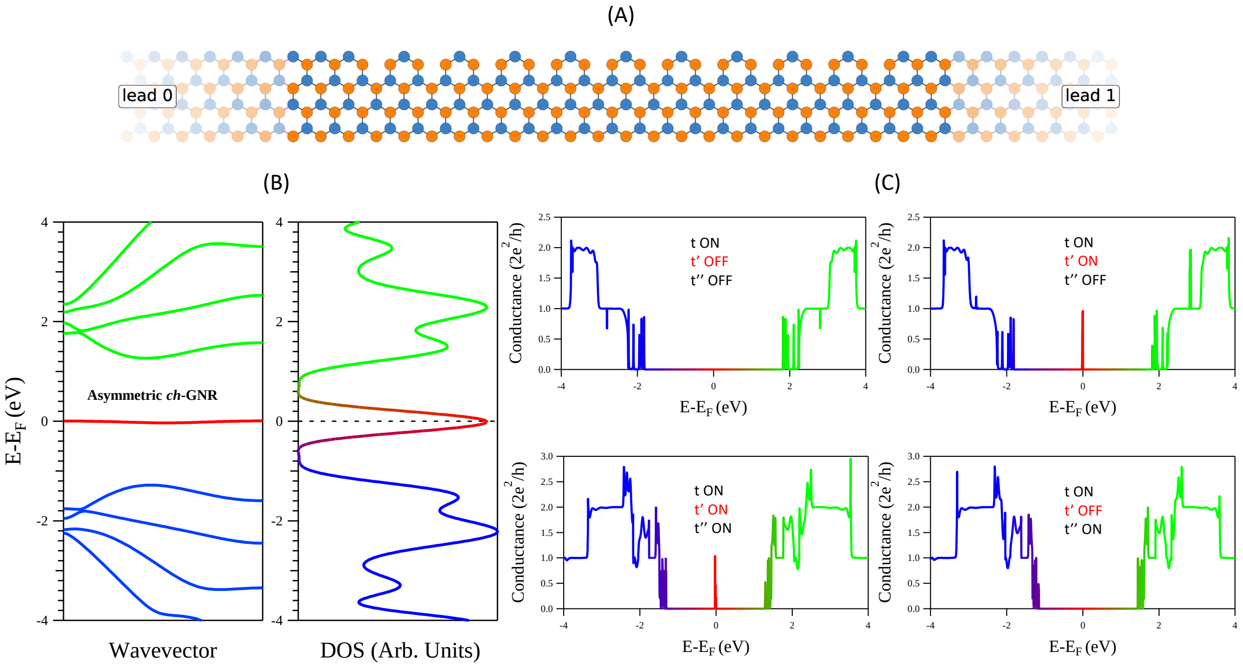

FIG. S5. Electronic structure and conductance in asymmetrically notched 4ZGNR: Asymmetric *ch*-GNR. (A) TB models of notched 4ZGNR with leads attached (B) TB calculated band structure (left) and the corresponding DOS (right) for asymmetric *ch*-GNR polymer, revealing the formation of a metallic flat band and DOS feature (red) at the Fermi energy, inside the energy gap separating the valence (blue) and conduction (green) bands. (C) Conductance spectra with different combinations of hopping parameters. A new conductance channel opens up precisely at the Fermi energy solely when the second-nearest neighbour is turned on.

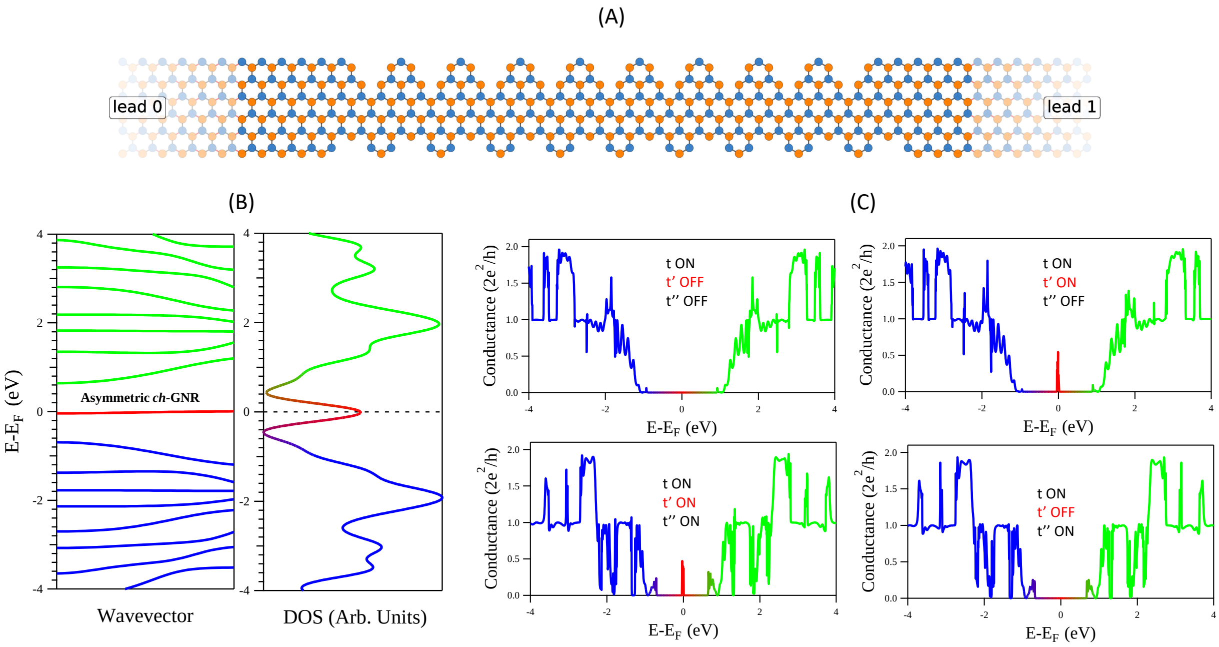

FIG. S6. Electronic structure and conductance in asymmetrically notched 6ZGNR: Asymmetric *ch*-GNR. (A) TB models of notched 6ZGNR with leads attached (B) TB calculated band structure (left) and the corresponding DOS (right) for asymmetric *ch*-GNR polymer, revealing the formation of a metallic flat band and DOS feature (red) at the Fermi energy, inside the energy gap separating the valence (blue) and conduction (green) bands. (C) Conductance spectra with different combinations of hopping parameters. A new conductance channel opens up precisely at the Fermi energy solely when the second-nearest neighbour is turned on.
